# Influence of genetic polymorphisms on omalizumab clinical response in pediatric severe asthma: a pilot study

**DOI:** 10.3389/fphar.2026.1857472

**Published:** 2026-07-21

**Authors:** Michelangelo Rottura, Igor Pirrotta, Chiara Cullotta, Ylenia Marino, Federica Maria Sacco, Viviana Maria Gianguzzo, Natasha Irrera, Vincenzo Arcoraci, Angela Alibrandi, Francesca Galletta, Marzia Corso, Salvatore Leonardi, Sara Manti, Giovanni Pallio

**Affiliations:** 1 Department of Clinical and Experimental Medicine, University of Messina, Messina, Italy; 2 Department of Biomedical and Dental Sciences and Morphological and Functional Imaging, University of Messina, Messina, Italy; 3 Department of Chemical, Biological, Pharmaceutical and Environmental Sciences, University of Messina, Messina, Italy; 4 Department of Human Pathology of Adult and Childhood Gaetano Barresi, University of Messina, Messina, Italy; 5 Pediatric Unit, University Hospital “Gaetano Martino”, Messina, Italy; 6 Pediatric Respiratory Unit, Department of Clinical and Experimental Medicine, San Marco Hospital, University of Catania, Catania, Italy

**Keywords:** biologic therapy, omalizumab, personalized medicine, pharmacogenetics, severe pediatric asthma, single nucleotide polymorphisms (SNPs)

## Abstract

**Introduction:**

Severe asthma affects 3%–5% of children and may remain uncontrolled despite high-dose inhaled corticosteroids and other controller therapies. Omalizumab, an anti-IgE monoclonal antibody, is approved for children ≥6 years with severe asthma, however treatment response shows substantial interindividual variability.

**Objectives:**

This study aimed to investigate whether selected single nucleotide polymorphisms (SNPs) in genes involved in IgE signaling and immune regulation are associated with clinical response to omalizumab in pediatric severe asthma.

**Methods:**

In this retrospective cohort study, 30 children with severe asthma treated with omalizumab for 12 months were genotyped for selected SNPs in *FCER1B*, *FCGR2A*, *FCGR3A*, *C3*, and *GATA2* genes. Clinical response was defined as: (i) ≥10% improvement in FEV1%, (ii) ≥50% reduction or absence of asthma exacerbations, and (iii) ≥50% reduction or discontinuation of oral corticosteroids (OCS). A combined response was defined as meeting at least two of these outcomes. Changes in GINA step were also assessed separately.

**Results:**

FEV1% improvement showed a nominal association with the variant allele of *GATA2* rs4857855 (p = 0.044). Reduction in exacerbation was nominally associated to variant alleles of *FCER1B* rs573790 (p = 0.003), and *GATA2* rs4857855 (p = 0.032). OCS reduction was nominally associated with variant alleles of *FCER1B* rs573790 (p = 0.037) and *FCGR3A* rs396991 (p = 0.014). A combined response was nominally associated with *FCER1B* rs573790 and *GATA2* rs4857855 variant alleles (p = 0.012). No SNP was associated with all three outcomes or GINA step reduction.

**Conclusion:**

Genetic variability in IgE- and Fc receptor–related pathways may contribute to differential response to omalizumab in children with severe asthma. These findings are exploratory and require validation in larger prospective studies.

## Introduction

Asthma remains one of the most prevalent and burdensome chronic respiratory diseases in children worldwide, affecting an estimated 10% of the global pediatric population and contributing substantially to morbidity, healthcare utilization, and diminished quality of life (Global Initiative for Asthma, 2024. Global Strategy for Asthma Management and Prevention, 2024). Although most cases are effectively managed with standard inhaled therapies, approximately 3%–5% of pediatric patients present with a severe, treatment-refractory form of the disease that remains uncontrolled despite high-dose inhaled corticosteroids (ICS), long-acting beta-agonists (LABA), and additional maintenance therapies (Manti et al., 2024). These patients account for a disproportionately high number of asthma-related exacerbations, hospitalizations, and healthcare costs (Pamuk et al., 2021), highlighting an urgent need to elucidate the underlying biological mechanisms to improve patient stratification and therapeutic decision-making (Chaverri Repáraz et al., 2025).

Pediatric severe asthma is a highly heterogeneous, multifactorial condition shaped by intricate interactions among genetic predisposition, immune dysfunction, and environmental exposures (Kondo et al., 2010). Recent advances in immunology have led to the introduction of biologic therapies that modulate key inflammatory pathways, particularly those associated with Type 2 (T2) inflammation (Anda Suárez et al., 2025). Omalizumab, a recombinant humanized monoclonal antibody targeting immunoglobulin E (IgE), was the first biologic therapy approved by both the U.S. Food and Drug Administration (FDA) and the European Medicines Agency (EMA) as an add-on treatment for patients aged ≥6 years with moderate-to-severe persistent allergic asthma, elevated serum IgE levels (30–1500 IU/mL), and sensitization to perennial aeroallergens (Food and Drug Administration, 2025; European Medicines Agency, 2025).

Omalizumab binds to free IgE and prevents its interaction with both high- (FcεRI) and low-affinity (FcεRII) receptors on mast cells, basophils, eosinophils, and dendritic cells, thereby downregulating receptor expression, inhibiting cellular activation and degranulation, and reducing the release of pro-inflammatory mediators (Jensen et al., 2015). Additional immunomodulatory effects include suppression of IgE synthesis, induction of apoptosis in effector cells, and enhancement of antiviral immunity (Foti Randazzese et al., 2025). Clinically, these mechanisms translate into reduced exacerbation rates, improved asthma control, decreased reliance on rescue medication, and enhanced quality of life for both children and their caregivers (Hanania et al., 2022; Kotoulas et al., 2022; Nagase et al., 2023; Caminiti et al., 2024).

Nevertheless, response to omalizumab is highly variable, and a substantial proportion of patients derive limited clinical benefit (Licari et al., 2019; Mobayed et al., 2025). While elevated baseline IgE levels and an allergic phenotype are associated with improved outcomes, these clinical indicators alone are insufficient to accurately predict treatment response (Lee et al., 2024). This heterogeneity has driven growing interest in the role of pharmacogenetics in modulating therapeutic efficacy (Rojo-Tolosa et al., 2023).

Variants in genes involved in IgE signaling (*FCER1A*, *FCER1B*), Fcγ receptor-mediated effector functions (*FCGR2A*, *FCGR3A*), innate immunity (*C3*), and immune cell differentiation (*GATA2*) are functionally relevant and may influence omalizumab responsiveness (Rojo-Tolosa et al., 2023). However, despite the growing emphasis on precision medicine, pharmacogenetic data in pediatric populations remain scarce and fragmented, particularly in the context of biologic therapies such as omalizumab**,** and no prospective studies have yet investigated these associations using a clinically integrated approach (Ferrante et al., 2022; Lim and Priefer, 2022).

To address this critical gap in pediatric asthma research, this study retrospectively evaluated whether selected single nucleotide polymorphisms (SNPs) in immunologically relevant genes are associated with clinical response to omalizumab in children with severe allergic asthma, aiming to identify early biomarkers to guide biologic treatment selection and advance precision medicine in pediatric asthma care.

## Materials and methods

### Study design

This retrospective, multicenter, observational study was conducted from April 2016 to April 2025 at the Pediatric Allergology Unit of the “G. Martino” Hospital, University of Messina, and the Pediatric Respiratory Unit of the San Marco Hospital, University of Catania, Italy.

The study included 30 pediatric patients (aged 6–18 years) of self-reported White European ancestry, of both sexes, with moderate-to-severe allergic asthma that remained uncontrolled despite optimized treatment with high doses of ICS-LABA. Inclusion criteria comprised: age > 6 years; a diagnosis of severe asthma confirmed according to current GINA guidelines (Global Initiative for Asthma, 2024. Global Strategy for Asthma Management and Prevention, 2024); and treatment with omalizumab for at least 12 months.

Demographic and clinical data were collected from medical records of the participating center according to routine clinical practice, including sex, age at initiation of omalizumab, baseline total serum IgE levels, and comorbidities (e.g., food allergies, obesity, allergic rhinitis, allergic conjunctivitis, and atopic dermatitis). Additional clinical and functional information included previous and concomitant treatments (oral corticosteroids (OCS), ICS, LABA, short-acting beta-agonists (SABA), number of asthma exacerbations, emergency department visits, hospitalizations, and forced expiratory volume in 1 s (FEV1%). These parameters were recorded for the 12 months prior to omalizumab initiation and during the 12-month treatment period.

To identify variables associated with treatment efficacy at 12 months, clinical response was defined by the following predefined outcomes: (1) a ≥50% reduction or complete discontinuation of annual OCS courses; (2) an increase of ≥10% in FEV1%; and (3) a ≥50% reduction or complete absence of asthma exacerbations requiring emergency department visits and/or hospitalization (Rojo-Tolosa et al., 2023; Del Mar Sánchez Suárez et al., 2025; Milger et al., 2025). We also explored whether specific patient characteristics were associated with achieving at least two or all three of these outcomes. In addition, a reduction of at least one step in the GINA treatment classification was also considered an indicator of treatment response (Beasley and Hatter, 2023).

### Ethics

All participants and their legal guardians received comprehensive information about the study and provided written informed consent in accordance with the approved study protocol. The study was conducted in compliance with Good Clinical Practice (GCP) guidelines and adhered to the ethical principles outlined in the Declaration of Helsinki and its subsequent amendments.

At the time of enrollment, clinicians provided a detailed explanation of the study objectives and procedures. Following informed consent, participants were asked to authorize the use of a portion of blood drawn for routine laboratory testing for the extraction of genomic DNA and the evaluation of genetic polymorphisms for research purposes. The study protocol was approved by the Ethics Committee of University Hospital Policlinico “G. Rodolico - San Marco” of Catania (approval number 280/2021, dated 01 March 2022).

### Sample collection and DNA extraction

Peripheral venous blood samples were collected using 3-mL tubes containing EDTA as anticoagulant and genomic DNA was extracted by using a standard phenol-chloroform protocol. Briefly, cells were first lysed in a DNA lysis buffer composed of 10 mM Tris-HCl pH 7.4, 100 mM NaCl, 1 mM EDTA, 1 mM MgCl_2_. After centrifugation, nuclear lysis was performed using 10% SDS and proteinase K, followed by overnight incubation at 37 °C. Proteins and other cellular debris were removed by phenol-chloroform extraction. The DNA was then washed with 70% ethanol and resuspended in RNase/DNase-free water. DNA concentration and purity were assessed spectrophotometrically using a NanoDrop® ND-1000 (Thermo Fisher Scientific, Wilmington, DE, USA). All DNA samples were stored at −20 °C until used for genotyping analysis (Pallio et al., 2020; Rottura et al., 2025).

### SNPs selection and genotyping

SNPs were selected using a candidate-gene approach based on their previously reported or biologically plausible involvement in IgE-mediated pathways, Fc receptor signaling, immune regulation, and mechanisms relevant to the pharmacodynamics of omalizumab (Gudbjartsson et al., 2009; Potaczek et al., 2013). Moreover, a previous pharmacogenetic study by Rojo-Tolosa et al., 2023already investigated the same SNPs in adult patients treated with omalizumab (Rojo-Tolosa et al., 2023). Therefore, the following six SNPs were analyzed: FCER1A rs2251746, FCER1B rs573790, FCGR2A rs1801274, FCGR3A rs396991, C3 rs2230199, and GATA2 rs4857855.

Genotyping was performed using predesigned TaqMan® assays (Thermo Fisher Scientific, Waltham, MA, USA) according to the manufacturer’s instructions. PCR reactions were set up in 96-well plates with a total volume of 25 μL per well, including 12.5 µL of 2× TaqMan Genotyping Master Mix, 1.25 µL of 20× assay mix, 1 µL of genomic DNA (10 ng/μL), and 10.25 µL of nuclease-free water. Plates were sealed and briefly centrifuged to consolidate contents and remove air bubbles.

PCR amplification was carried out using the QuantStudio™ 6 Flex Real-Time PCR System (Applied Biosystems, Foster City, CA, USA) under the following thermal cycling conditions: 95 °C for 10 min, followed by 40 cycles of 95 °C for 15 s and 60 °C for 1 min. Allelic discrimination analysis was conducted using QuantStudio™ software.

Each genotype was independently evaluated by two investigators. In the event of discordant calls, a third reviewer resolved the discrepancy. Subjects homozygous for the major allele were classified as wild type (WT).

### Statistical analysis

A descriptive analysis of the demographic and clinical characteristics of the enrolled patients was performed. Categorical variables were reported as absolute frequencies and percentages, while continuous variables were expressed as medians and interquartile ranges (Q1–Q3). The Kolmogorov–Smirnov test was used to assess the normality of data distribution; since some of the numerical variables were not normally distributed, a non-parametric approach was adopted. Hardy–Weinberg equilibrium for each SNP was assessed using the exact test.

The Mann–Whitney U test for independent samples and two-tailed Pearson chi-squared test were applied to compare two groups of subjects (Homo/Hete vs. WT) with reference to continuous and categorical variables, respectively.

The percentage change after 12 months of treatment was calculated for each of the predefined outcomes. These changes were further analyzed according to genotype, comparing individuals carrying the variant allele (heterozygous or homozygous) with WT individuals for each SNP. A p-value of <0.05 was considered statistically significant. Given the exploratory design and limited sample size, no formal correction for multiple testing was applied; therefore, all p-values should be interpreted as nominal. All statistical analyses were performed using SPSS Statistics version 23.0 (IBM Corp., SPSS Statistics, Armonk, NY, USA).

## Results

### Patients characteristics

A total of 30 pediatric patients were enrolled in the study. The median age was 12 years (IQR, 8–14), and the majority were male (n = 19; 63.3%). The most frequently reported comorbidities were atopic dermatitis (n = 13; 43.3%), allergic rhinitis (n = 13; 43.3%), allergic rhinoconjunctivitis (n = 10; 33.3%), and food or other allergies (n = 5; 16.7%).

The median baseline total serum IgE level at the initiation of omalizumab therapy was 321 IU/mL (IQR, 158–516). According to the Global Initiative for Asthma, 2024 classification at the start of biologic therapy, most patients were classified at step 2 or 3 (n = 12 each; 40.0%), with five (16.7%) at step 1 and one (3.3%) at step 4.

During the 12 months preceding omalizumab initiation, all patients had received at least one course of OCS and experienced at least one asthma exacerbation. Emergency department (ED) visits were required in 23 patients (76.7%), and 16 (53.3%) were hospitalized. At baseline, 9 patients (30.0%) had an FEV1% of less than 80% (Table 1).

**TABLE 1:** Patient’s characteristics.

Characteristic	Patients N = 30
Age; median (Q1-Q3)	12 (8–14)
Gender; n (%)	
Male	19 (63.3)
Female	11 (36.7)
Comorbidites; n (%)	
Atopic dermatitis	13 (43.3)
Allergic rhinitis	13 (43.3)
Allergic conjunctivitis	10 (33.3)
Allergy	5 (16.7)
Obesity	3 (10.0)
Epilepsy	1 (3.3)
Type 1 diabetes	1 (3.3)
Baseline IgE (IU/mL) median (Q1-Q3)	321 (158–516)
Step GINA; n (%)	
1	5 (16.7)
2	12 (40.0)
3	12 (40.0)
4	1 (3.3)
OCS courses; n (%)	
Yes	30 (100.0)
No	0 (0.0)
Asthma exacerbations; n (%)	
Yes	30 (100.0)
No	0 (0.0)
ED visits; n (%)	
Yes	23 (76.7)
No	7 (23.3)
Ordinary hospitalization; n (%)	
Yes	16 (53.3)
No	14 (46.7)
Baseline FEV1%; n (%)	
<80	9 (30.0)
>80	21 (70.0)

IgE: Immunoglobulin E; GINA: global initiative for asthma, 2024; OCS: oral corticosteroids; ED: emergency department; FEV1%: forced expiratory volume in 1 s.

### Distribution of the genotypes analyzed

All patients were genotyped for the six selected SNPs: *FCER1A* (rs2251746), *FCER1B* (rs573790), *C3* (rs2230199), *FCGR2A* (rs1801274), *FCGR3A* (rs396991), and *GATA2* (rs4857855). Genotype distributions were evaluated for conformity with Hardy–Weinberg equilibrium.

The genotype distribution for *FCER1A* rs2251746 deviated from Hardy–Weinberg equilibrium, whereas the remaining SNPs showed genotype distributions compatible with Hardy–Weinberg equilibrium. Given the small sample size and the disease-selected nature of the cohort, this deviation was not considered sufficient to exclude the variant from analyses (Table 2).

**TABLE 2:** Hardy-Weinberg equilibrium for the SNPs included in the study.

Gene	SNP	Genotype frequency			Observed heterozygosity	Expected heterozygosity	HWE
FCER1A	rs2251746	T/T	T/C	C/C	6.7%	49.1%	<0.001
		16 (53.3%)	2 (6.7%)	12 (40.0%)			
FCER1B	rs573790	C/C	C/T	T/T	33.3%	32.0%	1.000
		19 (63.4%)	10 (33.3%)	1 (3.3%)			
C3	rs2230199	G/G	G/C	C/C	36.7%	37.5%	1.000
		2 (6.7%)	11 (36.7%)	17 (56.7%)			
FCGR2A	rs1801274	A/A	A/G	G/G	46.7%	46.4%	1.000
		12 (40.0%)	14 (46.7%)	4 (13.3%)			
FCGR3A	rs396991	A/A	A/C	C/C	53.3%	49.1%	1.000
		5 (16.7%)	16 (53.3%)	9 (30.0%)			
GATA2	rs4857855	C/C	C/T	T/T	26.7%	35.8%	0.158
		19 (63.3%)	8 (26.7%)	3 (10.0%)			

### Association between genotype and FEV1% response

Twelve patients (40.0%) met the predefined criterion for FEV1% response, defined as a ≥10% increase in FEV1% after 12 months of omalizumab treatment (Table 1).

No statistically significant differences in the percentage change in FEV1% were observed between WT individuals and variant allele carriers for most of the analysed SNPs. Nevertheless, carriers of the variant allele for *GATA2* rs4857855, *FCGR3A* rs396991 and *FCGR2A* rs1801274, showed numerically greater median improvements in FEV1% compared with WT individuals, although these differences did not reach statistical significance.

**FIGURE 1:**
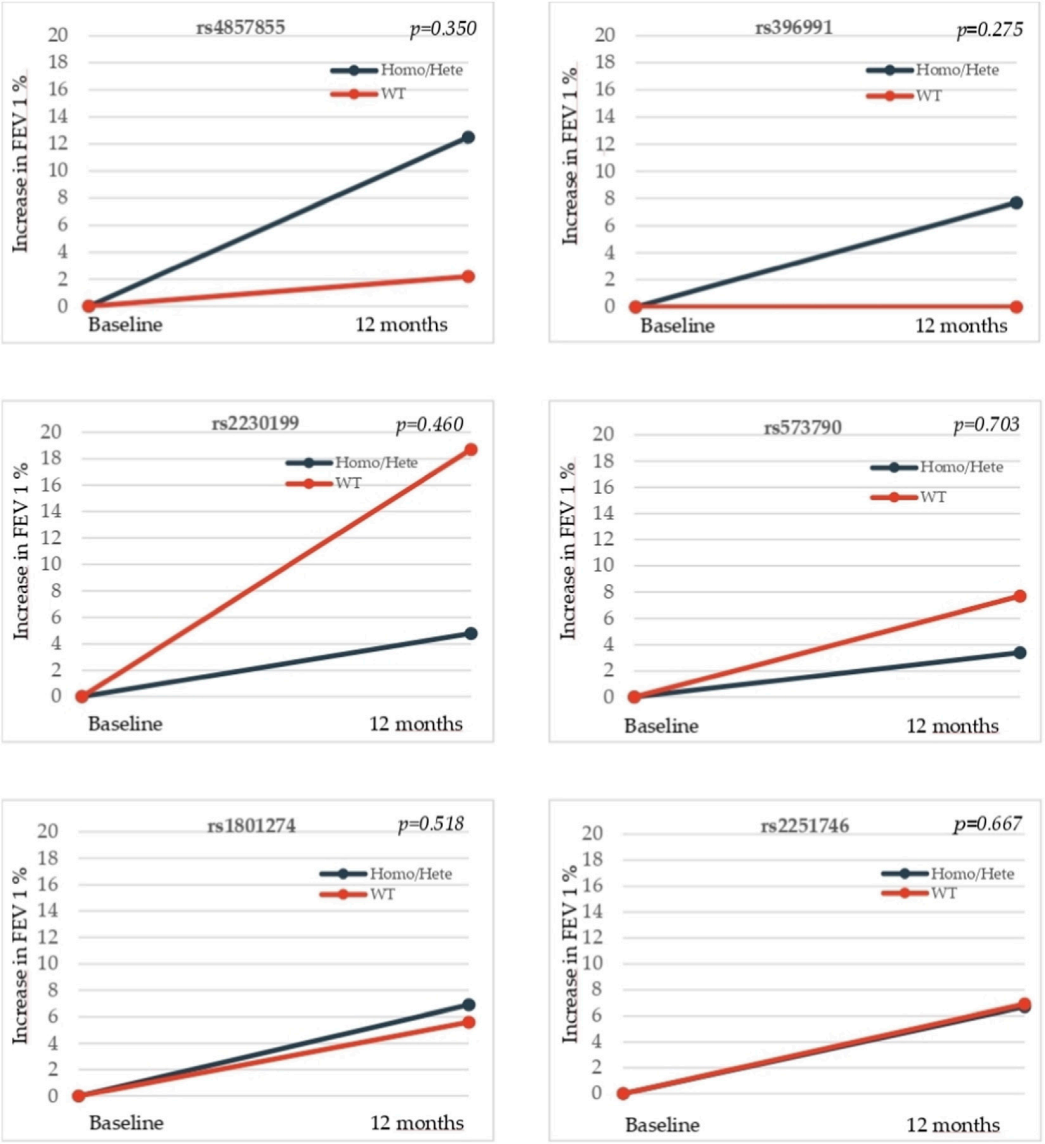
Percentage change in FEV1% after 12 months of omalizumab treatment by genotype. Median percentage change in FEV1%_1_ observed in wild-type (WT) versus variant allele carriers for each SNP. Statistical comparisons performed using the Mann–Whitney U test.

Conversely, WT individuals for *FCER1A* rs2251746, *FCER1B* rs573790 and *C3* rs2230199 exhibited numerically FEV1% improvements than variant carriers (Figure 1).

A nominal association was observed between carriage of the *GATA2* rs4857855 variant allele and FEV1% response (p = 0.044; Table 3), whereas none of the other SNPs showed statically significant associations.

**TABLE 3:** Association between SNP genotypes and FEV1% response.

SNPs	Genotype	n	FEV1%		P value
			NR 18	R 12	
rs4857855					
​	HOMO/HETE	11	4 (36.4)	7 (63.6)	0.044
​	WT	19	14 (73.7)	5 (26.3)	​
rs396991					
​	HOMO/HETE	25	14 (56.0)	11 (44.0)	0.317
​	WT	5	4 (80.0)	1 (20.0)	​
rs2230199					
​	HOMO/HETE	28	18 (64.3)	10 (35.7)	0.073
​	WT	2	0 (0.0)	2 (100.0)	​
rs573790					
​	HOMO/HETE	11	7 (63.6)	4 (36.4)	0.757
​	WT	19	11 (57.9)	8 (42.1)	​
rs1801274					
​	HOMO/HETE	18	11 (61.1)	7 (38.9)	0.879
​	WT	12	7 (58.3)	5 (41.7)	​
rs2251746					
​	HOMO/HETE	14	7 (50.0)	7 (50.0)	0.296
​	WT	16	11 (68.8)	5 (31.2)	​

FEV1%: forced expiratory volume in 1 s; NR: No-responder; R: responder; SNPs: Single nucleotide polymorphisms

### Association between genotype and exacerbation reduction response

Twenty patients (60.0%) were classified as responders for exacerbation reduction, defined as a ≥50% decrease in the annual number of exacerbations after 12 months of omalizumab treatment (Table 1).

**FIGURE 2:**
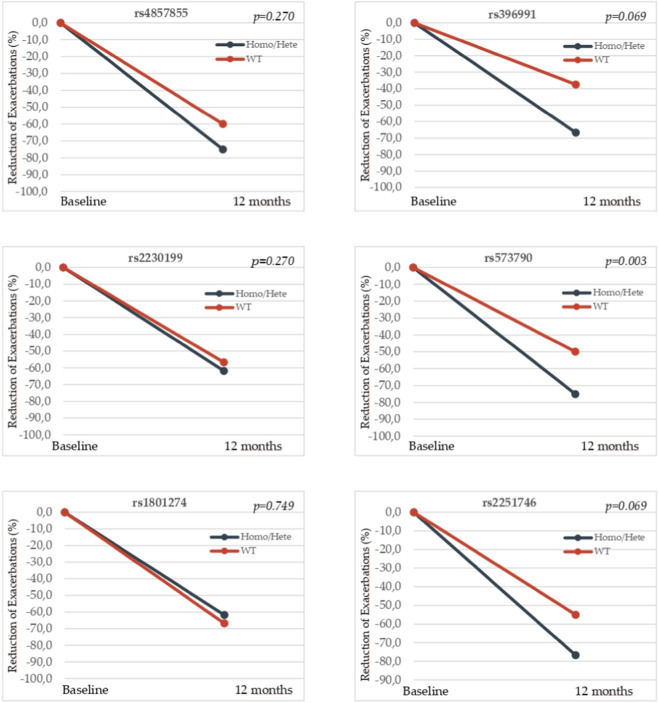
Percentage reduction in asthma exacerbations after 12 months of omalizumab treatment by genotype. Median percentage reduction in exacerbations observed in wild-type (WT) versus variant allele carriers for each SNP. Statistical comparisons performed using the Mann–Whitney U test.

Across all analysed SNPs, a numerically greater reduction in exacerbations was observed among variant allele carriers compared to WT individuals, except for *FCGR2A* rs1801274, where WT individuals showed a more favourable response. A statistically significant difference in percentage reduction was observed for *FCER1B* rs573790, with variant carriers showing a median reduction of 75.0% (IQR 72.2%–100.0%) versus 50.0% (IQR 37.0%–63.0%) in WT patients (p = 0.003; Figure 2).

Additionally, carriage of the variant allele in *FCER1A* rs2251746, *FCER1B* rs573790 and *GATA2* rs4857855 was nominally associated with a higher likelihood of response: the proportion of responders was numerically greater among variant carriers compared to WT subjects (rs2251746: 85.7% vs. 50.0%, p = 0.038; rs573790: 100.0% vs. 47.4%, p = 0.003; rs4857855: 90.9% vs. 52.6%, p = 0.032; Table 4).

**TABLE 4:** Association between SNPs genotypes and exacerbation reduction.

SNPs	Genotype	n	Exacerbations reduction		P value
			NR 10	R 20	
rs4857855					
​	HOMO/HETE	11	1 (9.1)	10 (90.9)	0.032
​	WT	19	9 (47.4)	10 (52.6)	​
rs396991					
​	HOMO/HETE	25	7 (28.0)	18 (72.0)	0.166
​	WT	5	3 (60.0)	2 (40.0)	​
rs2230199					
​	HOMO/HETE	28	10 (35.7)	18 (64.3)	0.301
​	WT	2	0 (0.0)	2 (100.0)	​
rs573790					
​	HOMO/HETE	11	0 (0.0)	11 (100.0)	0.003
​	WT	19	10 (52.6)	9 (47.4)	​
rs1801274					
​	HOMO/HETE	18	8 (44.4)	10 (55.6)	0.114
​	WT	12	2 (16.7)	10 (83.3)	​
rs2251746					
​	HOMO/HETE	14	2 (14.3)	12 (85.7)	0.038
​	WT	16	8 (50.0)	8 (50.0)	​

NR: No-responder; R: responder; SNPs: Single nucleotide polymorphisms

### Association between genotype and OCS reduction response

Twenty-four patients (80.0%) met the response criterion for OCS reduction, defined as a ≥50% decrease in the annual number of OCS courses after 12 months of omalizumab treatment (Table 1).

**FIGURE 3:**
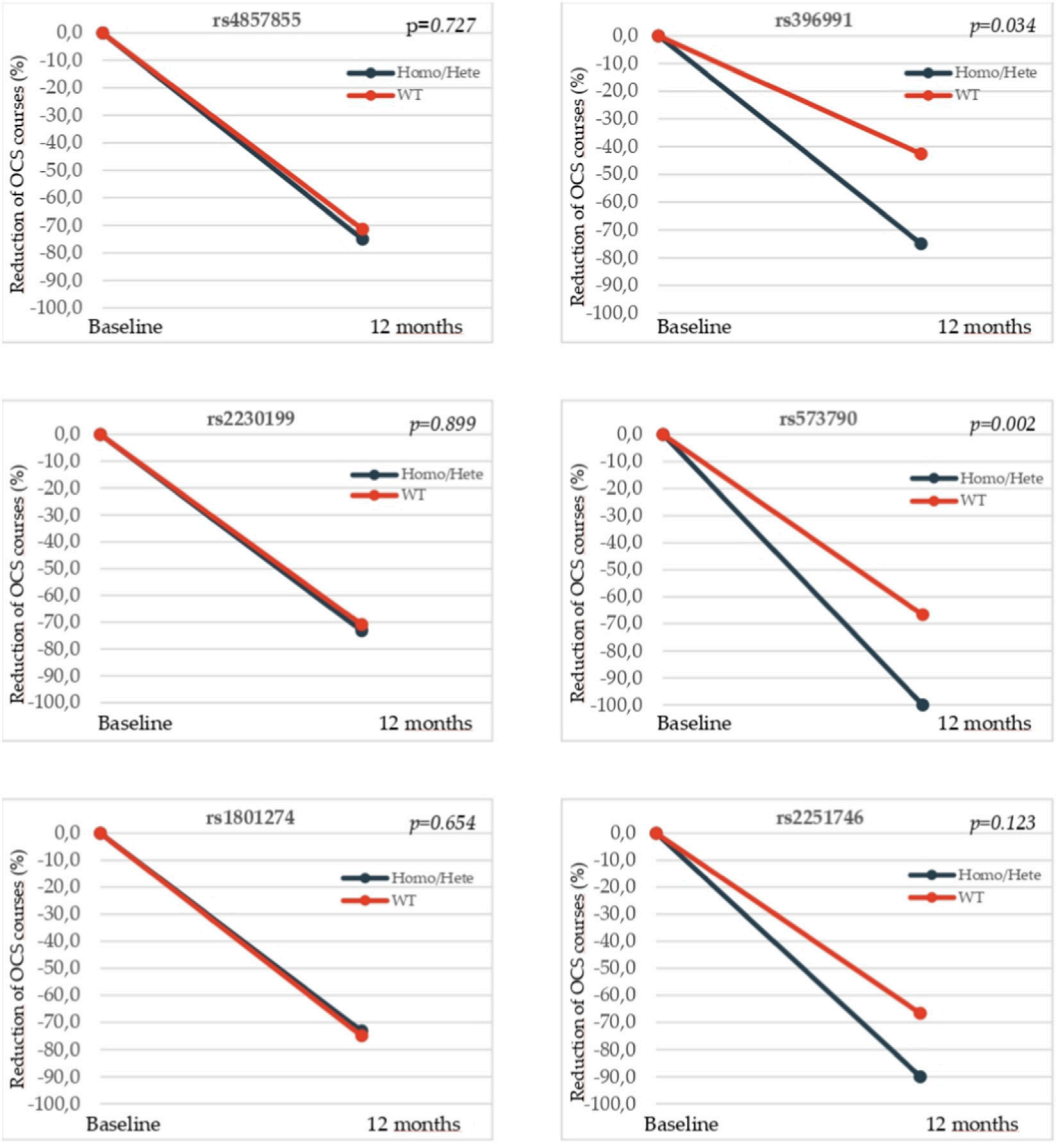
Percentage reduction in OCS courses after 12 months of omalizumab treatment by genotype. Median percentage reduction in the number of OCS courses observed in wild-type (WT) versus variant allele carriers for each SNP. Statistical comparisons performed using the Mann–Whitney U test.

Across all analyzed SNPs, a numerically greater reduction in OCS use was observed among variant allele carriers compared to WT individuals, except for *FCGR2A* rs1801274, where WT subjects demonstrated a more favourable response. Statistically significant differences were found for *FCGR3A* rs396991 and *FCER1B* rs573790. Carriers of the rs396991 variant showed a median reduction of −75.0% (IQR –66.7% to −100.0%) versus −42.6% (IQR –16.7% to −80.0%) in WT patients (p = 0.034), while those with the rs573790 variant exhibited a reduction of −100.0% (IQR –80.0% to −100.0%) compared to −66.7% (IQR –50.0% to −75.0%) in the WT group (p = 0.002; Figure 3).

Additionally, carriage of the variant allele in rs573790 and rs396991 was nominally associated with a higher likelihood of response: 100.0% vs. 68.4% for rs573790 (p = 0.037) and 88.0% vs. 40.0% for rs396991 (p = 0.014; Table 5).

**TABLE 5:** Association between SNPs genotypes and OCS reduction.

SNPs	Genotype	n	OCS reduction		P value
			NR 6	R 24	
rs4857855					
​	HOMO/HETE	11	2 (18.2)	9 (81.8)	0.850
​	WT	19	4 (21.1)	15 (78.9)	​
rs396991					
​	HOMO/HETE	25	3 (12.0)	22 (88.0)	0.014
​	WT	5	3 (60.0)	2 (40.0)	​
rs2230199					
​	HOMO/HETE	28	5 (17.9)	23 (82.1)	0.272
​	WT	2	1 (50.0)	1 (50.0)	​
rs573790					
​	HOMO/HETE	11	0 (0.0)	11 (100.0)	0.037
​	WT	19	6 (31.6)	13 (68.4)	​
rs1801274					
​	HOMO/HETE	18	4 (22.2)	14 (77.8)	0.709
​	WT	12	2 (16.7)	10 (83.3)	​
rs2251746					
​	HOMO/HETE	14	3 (21.4)	11 (78.6)	0.855
​	WT	16	3 (18.8)	13 (81.2)	​

NR: No-responder; OCS: oral corticosteroids; R: responder; SNPs: Single nucleotide polymorphisms

### Multi-outcome response analysis

Twenty-two patients (73.3%) were classified as responders in at least two outcome domains (Table 6). A numerically higher proportion of multi-outcome responders was observed among variant allele carriers of *GATA2* rs4857855 and *FCER1B* rs573790 (100.0% vs. 57.9%, p = 0.012; Table 6), suggesting a potential association between these polymorphisms and broader treatment responsiveness.

**TABLE 6:** Association between SNPs genotypes and response in multiple outcomes.

SNPs	Genotype	n	Two outcomes		P value	Three outcomes		P value
			NR 8	R 22		NR 22	R 8	
rs4857855								
​	HOMO/HETE	11	0 (0.0)	11 (100.0)	0.012	7 (63.6)	4 (36.4)	0.361
​	WT	19	8 (42.1)	11 (57.9)	​	15 (78.9)	4 (21.1)	​
rs396991								
​	HOMO/HETE	25	5 (20.0)	20 (80.0)	0.065	17 (68.0)	8 (32.0)	0.140
​	WT	5	3 (60.0)	2 (40.0)	​	5 (100.0)	0 (0.0)	​
rs2230199								
​	HOMO/HETE	28	8 (28.6)	20 (71.4)	0.377	21 (75.0)	7 (25.0)	0.440
​	WT	2	0 (0.0)	2 (100.0)	​	1 (50.0)	1 (50.0)	​
rs573790								
​	HOMO/HETE	11	0 (0.0)	11 (100.0)	0.012	7 (63.6)	4 (36.4)	0.361
​	WT	19	8 (42.1)	11 (57.9)	​	15 (78.9)	4 (21.1)	​
rs1801274								
​	HOMO/HETE	18	6 (33.3)	12 (66.7)	0.312	14 (77.8)	4 (22.2)	0.500
​	WT	12	2 (16.7)	10 (83.3)	​	8 (66.7)	4 (33.3)	​
rs2251746								
​	HOMO/HETE	14	2 (14.3)	12 (85.7)	0.151	9 (64.3)	5 (35.7)	0.295
​	WT	16	6 (37.5)	10 (62.5)	​	13 (81.2)	3 (18.8)	​

NR: No-responder; R: responder; SNPs: Single nucleotide polymorphisms

Only eight patients (26.7%) met response criteria across all three outcomes. However, no statistically significant associations were observed for any of the SNPs analysed (Table 6).

### Association between genotype and clinical improvement by GINA step

**FIGURE 4:**
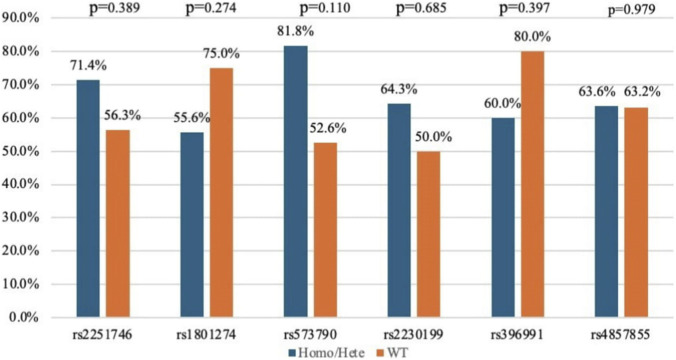
Clinical improvement by genotype assessed via GINA step. Percentage of patients achieving a reduction of at least one GINA treatment step after 12 months of omalizumab therapy, stratified by genotype for each SNP. Statistical comparisons performed using the Pearson chi-squared test.

Carriers of the variant alleles in *FCER1A* rs2251746 and *FCER1B* rs573790 exhibited a higher proportion of clinical improvement defined as a reduction of at least one GINA treatment step after 12 months compared to WT patients (71.4% vs. 56.3% and 81.8% vs. 52.6%, respectively). Conversely, clinical improvement was more frequently observed among WT individuals for *FCGR2A* rs1801274 (75.0% vs. 55.6%) and *FCGR3A* rs396991 (80.0% vs. 60.0%). However, none of these differences reached statistical significance (Figure 4).

## Discussion

In this study, we investigated whether selected candidate genetic variants involved in IgE signaling and immune effector pathways were associated with clinical response to omalizumab in a pediatric cohort with severe asthma. Given the limited sample size, our findings should be interpreted as exploratory and hypothesis-generating, suggesting that variability in genes related to Fc receptor signaling and immune regulation may contribute to interindividual differences in treatment response.

The mechanism of action of omalizumab is based on binding circulating free IgE and downregulating the expression of high-affinity IgE receptors (FcεRI) on mast cells, basophils, and dendritic cells, thereby attenuating IgE-mediated allergic inflammation (Domingo et al., 2024). Genetic variability within components of this pathway may influence receptor expression, immune cell activation, and downstream clinical effects, potentially modulating treatment efficacy (Rojo-Tolosa et al., 2023).

In this context, although FCER1A rs2251746 showed a marked deviation from Hardy–Weinberg equilibrium in our study cohort, the *FCER1A* rs2251746 variant allele was nominally associated with a reduction in asthma exacerbation in our cohort. This partially contrasts with a preceding paper, which reported a higher probability of achieving general response criteria in T allele carriers but did not identify a specific effect on exacerbation reduction (Rojo-Tolosa et al., 2023). In 72 White adult patients with severe eosinophilic asthma, the same authors did not find significant associations between this SNP and treatment response to mepolizumab or benralizumab (Rojo-Tolosa et al., 2024). Moreover, in a study conducted in 100 patients with chronic spontaneous urticaria, no significant relationship was found between rs2251746 polymorphism and resistance to omalizumab therapy (Savas et al., 2024), nor was any association found between omalizumab drug survival and this SNP in 110 patients with uncontrolled severe allergic asthma (Rojo-Tolosa et al., 2025). This variant influences the expression of the α subunit of the high-affinity IgE receptor, which amplifies FcεRI signaling and plays a central role in allergic inflammation due to its association with high serum IgE levels (Granada et al., 2012; Zhou et al., 2012; Potaczek et al., 2013; Liao et al., 2015), which have already been identified as a predictor of response to omalizumab therapy in children with persistent asthma and greater clinical improvement (Sheehan et al., 2020), thus supporting the protective role of this SNP, as identified in our study.

Variants in *FCER1B*, which encodes the β subunit of FcεRI, emerged as the most consistently associated genetic signals across multiple clinical outcomes in our cohort.

In particular, *FCER1B* rs573790 showed nominal associations with reduced exacerbation frequency, decreased use of OCS, and response across at least two clinical domains. These observations partially contrast with findings by Rojo-Tolosa et al. (2023), who reported a higher probability of response among WT individuals for exacerbation frequency, and no associations for other clinical outcomes in adults treated with omalizumab (Rojo-Tolosa et al., 2023). Similarly, no significant associations were observed between rs573790 and treatment response to other biologics in adults with asthma (Rojo-Tolosa et al., 2024). Conversely, the TT genotype has been associated with longer omalizumab treatment persistence in patients with atopic asthma (Rojo-Tolosa et al., 2025). Functionally, rs573790 affects the β subunit of FcεRI and has been linked to high total IgE levels (HIZAWA et al., 2000), providing a biologically plausible rationale supporting our results.

The *FCGR3A* rs396991 variant showed a nominal association with reduced OCS use. This finding partially aligns previous evidence showing improved lung function among variant carriers treated with omalizumab (Rojo-Tolosa et al., 2023), although effects on OCS use were not previously reported. Beyond asthma, rs396991 has been implicated in response to anti-tumour necrosis factor α (TNFα) therapy in rheumatoid arthritis (Cañete et al., 2009), reflecting the role of FcγRIIIa in immune complex handling and antibody-dependent cellular cytotoxicity (ADCC). These mechanisms may influence omalizumab pharmacokinetics and clearance, potentially contributing to variability in short-term efficacy versus long-term persistence, as suggested by prior drug survival studies (Rojo-Tolosa et al., 2025). Notably, no associations between this SNP and response to mepolizumab or benralizumab have been observed in adult patients with severe asthma (Rojo-Tolosa et al., 2024), supporting a possible drug-specific effect related to omalizumab pharmacodynamics.

Variant in *GATA2* rs4857855 was nominally associated with improvements in lung function, reduced exacerbation frequency, and multi-domain clinical response. These findings are consistent with those reported by Rojo-Tolosa et al. (2023) in adult patients receiving omalizumab (Rojo-Tolosa et al., 2023). GATA2 plays a central role in hematopoietic differentiation and immune regulation, and this SNP has previously been linked to asthma susceptibility and eosinophilic inflammation (Gudbjartsson et al., 2009). The absence of similar associations in patients treated with other biologics or in omalizumab drug survival analyses (Rojo-Tolosa et al., 2024; Rojo-Tolosa et al., 2025), further suggests a potential specificity of this variant for anti-IgE therapy, although confirmation in larger pediatric cohorts is required.

In contrast, *FCGR2A* rs1801274, was not associated with any clinical endpoints in our pediatric population (Wu et al., 2014). These findings are consistent with previous negative results regarding response to mepolizumab, benralizumab, and omalizumab persistence (Rojo-Tolosa et al., 2024; Rojo-Tolosa et al., 2025), although earlier work reported improved lung function among variant carriers treated with omalizumab (Rojo-Tolosa et al., 2023). Similarly, *C3* rs2230199, despite its role in complement activation and airway inflammation, was not associated with treatment response in our study, in line with omalizumab drug survival data (Rojo-Tolosa et al., 2025), but differing from a previous paper linking the C allele to exacerbation reduction in adults with severe asthma treated with omalizumab (Rojo-Tolosa et al., 2023). These discrepancies highlight the heterogeneity of pharmacogenetic effects across populations, age groups, and clinical endpoints.

From a multidomain perspective, variant alleles in *GATA2* rs4857855 and *FCER1B* rs573790 were nominally associated with response across at least two clinical outcomes suggesting that integrative response definitions may better capture treatment benefit than single endpoints. However, no genetic variant was associated with response across all three outcomes, underscoring the multifactorial nature of asthma and the limitations of relying on single genetic markers.

Changes in GINA treatment step were also explored as a surrogate for clinical improvement. Although numerically favorable trends were observed for some variants, none of the associations reached statistical significance. This may reflect the semi-quantitative nature of step-down assessment and the limited sensitivity of this outcome in small cohorts.

The clinical relevance of pharmacogenetics in guiding biologic therapy is increasingly recognized, especially in pediatric asthma, where treatment options are limited, and evidence is often extrapolated from adult population. While genome-wide association studies (GWAS) remain scarce in this field, our findings support the continued investigation of candidate gene approaches focused on biologically relevant pathways, such as IgE signalling. If validated, pharmacogenetic profiling may contribute to earlier identification of likely responders and efficient allocation of biologic therapies.

## Strengths and limitations

This study has several limitations that should be carefully considered when interpreting the findings.

First, the relatively small sample size limits statistical power and increases the likelihood of spurious associations, particularly in the absence of large-scale validation cohorts, and reduces the ability to detect modest effects and associations involving less frequent alleles. This constraint also precluded the application of formal corrections for multiple testing, and therefore all reported p-values should be regarded as nominal and hypothesis-generating. Larger cohorts will be necessary to confirm the observed trends and to enable more robust statistical modeling.

Second, multiple SNPs and outcomes were tested without correction for multiple comparisons, increasing the risk of type I error and false-positive findings. Therefore, all reported associations should be interpreted as nominal and require confirmation in larger studies.

Third, the genotype distribution of FCER1A rs2251746 deviated from Hardy–Weinberg equilibrium. Although genotyping quality was independently reviewed and no technical inconsistencies were identified, the observed departure from Hardy–Weinberg equilibrium is likely attributable to the very small sample size of the present study. Therefore, any association involving this SNP should be interpreted with particular caution and regarded as particularly tentative until confirmed in larger, independent, and appropriately powered cohorts. Fourth, the retrospective design may have introduced selection and information bias, as clinical data were collected from medical records rather than through standardized prospective protocols. In addition, detailed molecular and inflammatory biomarkers, such as fractional exhaled nitric oxide (FeNO) or longitudinal eosinophil measurements, were not systematically available, limiting the ability to integrate genetic findings with endotype-specific features of severe asthma.

Fifth, ancestry was determined based on clinical records and self-reported information rather than through genetic inference or principal component analysis. Although all participants were classified as of European ancestry, subtle population stratification cannot be completely excluded and may have influenced genotype–phenotype associations. Furthermore, gene–environment interactions, epigenetic regulation, and other layers of biological complexity that are known to contribute to asthma heterogeneity were not assessed in this study.

Despite these limitations, the study also has several important strengths. To our knowledge, this is the first investigation specifically addressing the pharmacogenetics of omalizumab in a pediatric population with severe asthma, a group for whom treatment decisions are often extrapolated from adult data. The focus on children therefore addresses a relevant and underexplored clinical need.

Another strength lies in the candidate-gene approach, which targeted polymorphisms in biologically plausible pathways directly related to IgE signaling, Fc receptor function, and immune regulation. This hypothesis-driven strategy reduces the likelihood of spurious findings compared with agnostic approaches in small cohorts and enhances the biological interpretability of the observed associations.

In addition, multiple clinically meaningful outcomes were evaluated, including lung function, exacerbation frequency, oral corticosteroid use, and multidomain response definitions. This comprehensive assessment provides a more nuanced characterization of treatment response than reliance on a single endpoint and reflects real-world clinical decision-making in pediatric severe asthma.

Finally, the real-world setting of this study increases the clinical relevance of the findings, as patients were managed according to routine practice rather than highly controlled trial conditions. Although this may increase variability, it enhances the generalizability of the observations to everyday clinical contexts.

Taken together, while the results of this study should be interpreted with caution, the combination of biological plausibility, multidomain outcome assessment, and pediatric focus supports the value of these findings as a foundation for future prospective and multicenter studies.

## Conclusion

This study provides exploratory, hypothesis-generating evidence that genetic variants in *FCER1A*, *FCER1B*, *FCGR3A*, and *GATA2* may contribute to interindividual variability in response to omalizumab in children with severe asthma. While preliminary, these findings highlight the potential clinical relevance of pharmacogenetic profiling and support the need for larger prospective studies to validate these observations before translation into clinical practice. In the future, integration of pharmacogenetic profiling into clinical decision-making may help enable more personalized treatment strategies and improve therapeutic outcomes in this vulnerable population.

## Data Availability

The original contributions presented in the study are included in the article/supplementary material, further inquiries can be directed to the corresponding author.
